# Urinary endogenous sex hormone levels in postmenopausal women after caloric restriction in young adulthood

**DOI:** 10.1038/sj.bjc.6601513

**Published:** 2004-01-06

**Authors:** S G Elias, N C Onland-Moret, P H M Peeters, S Rinaldi, R Kaaks, D E Grobbee, P A H van Noord

**Affiliations:** 1 Julius Center for Health Sciences and Primary Care, University Medical Center, PO Box 85500, Utrecht 3508 GA, The Netherlands; 2 Hormones and Cancer Group, International Agency for Research on Cancer, 150 Cours Albert Thomas, Lyon 69372, France

**Keywords:** caloric restriction, famine, sex hormones, parity, human

## Abstract

We investigated whether the 1944–1945 Dutch famine has affected postmenopausal sex hormone concentrations with data from 163 women (young adults during the famine). Urinary sex hormone concentrations showed modest elevations with increasing famine exposure. Effects were absent in parous women, but more pronounced in women who had never given birth.

In 1909, Moreschi was the first to report that caloric restriction inhibits the development of tumours in mice (Moreschi, 1909). This was subsequently found to be highly reproducible in other rodents, and to involve a variety of tumours (Weindruch and Walford, 1988). Already in the early days in this field of research, scientists recognised that caloric restriction might act via neuro-endocrine mechanisms to inhibit tumour initiation and promotion (Pomerantz and Mulinos, 1939). Since little is known about caloric restriction and cancer risk in humans, we investigated whether exposure to the 1944–1945 Dutch famine in young adulthood influenced urinary sex hormone concentrations in postmenopausal women. Given the lower risk of cancer in rodents fed a calorie-restricted diet, famine might be expected to be associated with lower endogenous sex hormone levels in women, since women with lower levels of such hormones have been found to have a decreased risk for breast cancer (The Endogenous Hormones and Breast Cancer Collaborative Group, 2002; Onland-Moret *et al*, 2003).

The famine occurred at the end of World War II in the densely populated western parts of the Netherlands with food supplies deteriorating rapidly from October 1944 onwards: official daily rations dropped from about 1500 kcal in September 1944 to below 700 kcal in January 1945. After 6 months of starvation, the Netherlands were liberated on 5 May 1945, abruptly ending the famine (Burger *et al*, 1948).

## SUBJECTS AND METHODS

As part of the Diagnostisch Onderzoek Mammacarcinoom (DOM) project, a population-based breast cancer-screening project was conducted between 1974 and 1986 in Utrecht, the Netherlands (de Waard *et al*, 1984), which from 1983 included questions about hunger, cold and weight loss experienced during the 1944–1945 Dutch famine, with answers to these questions as absent, moderate or severe exposure. We combined the answers into a three-level individual famine score as follows: women with severe or absent exposure to at least two aspects of famine (hunger, cold, or weight loss) were classified as severely or, respectively, unexposed to the famine. All the remaining women were classified as having been moderately exposed to the famine (Elias *et al*, 2002). Demographic details and reproductive history were also obtained by questionnaire. Anthropometrical details were collected at recruitment and participants donated a first morning urine sample on the day of their first mammographic examination. These urine samples were stored at −20°C in 250 ml plastic polypropylene jars, without preserving agents, until analysis. A random sample of 424 women with a natural menopause at recruitment and no history of breast cancer was drawn from 27 718 women, born between 1911 and 1945, as a control group for a study addressing the relation of endogenous sex steroids with postmenopausal breast cancer risk (Onland-Moret *et al*, 2003). At the introduction of the famine questionnaire in 1983, 203 women from this random sample still participated in the DOM cohort, of whom 182 resided in the occupied Netherlands during the famine and had sufficient data to compute the famine score. Women using hormone replacement therapy or oral contraceptives at the time of urine sampling were excluded (*n*=19).

Urine samples were analysed at the Hormones and Cancer Group, at the International Agency for Research on Cancer in Lyon, France. The hormones oestrone, oestradiol, testosterone and 5*α*-androstane-3*α*,17*β*-diol (3*α*D) were measured by radioimmunoassay after enzymatic hydrolysis, solid-phase extraction and high-performance liquid chromatography purification of the urine samples. The method used in this study and measures of its reproducibility have been described in detail elsewhere (Rinaldi *et al*, 2003). Intra- and interassay coefficients of variation were 8.7 and 17.2% for oestrone, 12.2 and 14.8% for oestradiol, 8.3 and 15.5% for testosterone and 9.0 and 11.4% for 3*α*D. Creatinine was measured in each sample by kinetic Jaffé reaction (Hitachi 717, Roche, Central Laboratory for Biochemistry, Hôpital de l'Antiquaille, Lyon, France). Hormone concentrations, expressed in ng per mg creatinine to adjust for the differences in urine dilution, were logarithmically transformed to achieve normal distributions. The mean hormone concentrations according to famine exposure categories were then estimated by analysis of covariance, and adjusted for potential confounders (age, body mass index, years since menopause, and cigarette smoking (yes/no) at the time of donation of the urine sample, as well as socio-economic status (high/low) and parity (parous/nulliparous)). Since pregnancy is known to alter – in some aspects permanently – the neuro-endocrine milieu (Bernstein, 2002), we tested whether any famine effects depended on parity. We used linear regression to evaluate the linear trends for the effect of famine exposure (quantitatively scored as 1, 2 or 3 with increasing exposure) and to test the interaction terms. Statistical analyses were performed with SPSS 11. All tests of statistical significance were two-sided. Backtransformation of the logarithmic means resulted in geometrical means, which we report together with the corresponding 95% confidence intervals (CIs).

## RESULTS

In total, 29 of the women were severely, 71 moderately and 63 unexposed to the famine. The median age during the famine was 26 years (10–90th percentile: 19–32 years). Overall, we found that famine was modestly associated with oestrone, with lower levels in the unexposed group compared to the moderately and severely exposed (*P*_trend_=0.07). The famine was apparently not related to oestradiol; levels of testosterone and 3*α*D were increased in the famine-exposed groups with highest levels in women with moderate famine exposures (Table 1

**Table 1:** Geometric means^a^ and 95% confidence intervals (CI) of oestrone, oestradiol, testosterone and 5*α*-androstane-3*α*,17*β*-diol levels in urine after correction for creatinine according to the famine score

	**Famine score**						
	**Unexposed (*n*=63)**		**Moderately exposed (*n*=71)**		**Severely exposed (*n*=29)**		
	**Geometric mean**	**95% CI**	**Geometric mean**	**95% CI**	**Geometric mean**	**95% CI**	
Oestrone	1.53	(1.32–1.78)	1.81	(1.57–2.08)	1.91	(1.54–2.38)	*P*_trend_=0.07^b^
Oestradiol	0.51	(0.44–0.59)	0.51	(0.44–0.58)	0.54	(0.44–0.67)	*P*_trend_=0.68
Testosterone	2.20	(1.81–2.67)	2.57	(2.14–3.08)	2.50	(1.88–3.33)	*P*_trend_=0.35
5*α*-androstane-3*α*,17*β*-diol	18.20	(15.10–21.94)	22.69	(19.02–27.07)	20.26	(15.39–26.66)	*P*_trend_=0.32

a Expressed in ng per mg creatinine and adjusted for age, body mass index, years since menopause and cigarette smoking at time of donation of urine sample as well as socio-economic status and parity.

b *P* for linear trend.

).

Parity seemed to modify the associations (interaction tests: oestrone: *P*=0.04; oestradiol: *P*<0.01; testosterone: *P*=0.09; 3*α*D: *P*=0.70). Famine was not related to any of the hormones in parous women, but showed some evidence of a dose–response relation with oestrone and oestradiol in women who never gave birth (Table 2

**Table 2:** Geometric means^a^ and 95% confidence intervals (CI) of estrone, estradiol, testosterone, and 5*α*-androstane-3*α*, 17*β*-diol levels in urine after correction for creatinine according to the famine score and parity

	**Famine score**						
	**Unexposed**		**Moderately exposed**		**Severely exposed**		
	**Geometric mean**	**95% CI**	**Geometric mean**	**95% CI**	**Geometric mean**	**95% CI**	
Nulliparous women	(*n*=10)		(*n*=20)		(*n*=6)		
Oestrone	1.24	(0.92–1.67)	1.61	(1.31–1.99)	3.36	(2.26–5.01)	*P*_trend_=0.001^b^
Oestradiol	0.37	(0.27–0.51)	0.57	(0.46–0.72)	0.91	(0.60–1.39)	*P*_trend_=0.001
Testosterone	2.04	(1.05–3.96)	2.51	(1.58–4.00)	4.19	(1.74–10.10)	*P*_trend_=0.21
5*α*-androstane-3*α*,17*β*-diol	24.98	(16.52–37.78)	20.88	(15.63–27.90)	29.80	(17.20–51.64)	*P*_trend_=0.78
Parous women	(*n*=53)		(*n*=51)		(*n*=23)		
Oestrone	1.60	(1.35–1.89)	1.87	(1.57–2.22)	1.69	(1.31–2.18)	*P*_trend_=0.51
Oestradiol	0.54	(0.45–0.63)	0.49	(0.41–0.58)	0.48	(0.37–0.62)	*P*_trend_=0.37
Testosterone	2.30	(1.89–2.79)	2.51	(2.05–3.05)	2.20	(1.64–2.95)	*P*_trend_=0.98
5*α*-androstane-3*α*,17*β*-diol	17.03	(13.85–20.95)	23.49	(19.01–29.04)	18.50	(13.52–25.33)	*P*_trend_=0.34

a Expressed in ng per mg creatinine and adjusted for age, body mass index, years since menopause and cigarette smoking at time of donation of urine sample as well as socio-economic status and parity.

b *P* for linear trend. Tests for linear interaction of famine exposure with parity: *P*=0.04 (oestrone); *P*<0.01 (oestradiol); *P*=0.09 (testosterone); *P*=0.70 (5*α*-androstane-3*α*,17*β*-diol).

). The association with testosterone showed the same tendency, although weaker. Inspection of the data revealed that outliers or between-batch variation did not explain the results.

## DISCUSSION

In conclusion, we found a dose–response increase of postmenopausal urinary oestrone and oestradiol levels with increasing severity of famine exposure in young adulthood among women who had never given birth, but not among parous women. Similar effects were seen for testosterone, whereas famine was not related to 3*α*D.

Although a black episode in the history of the Netherlands, the 1944–1945 Dutch famine has enabled us to investigate the long-term effects of a short but severe famine in an otherwise well-nourished human population. We were able to individually classify women in terms of their famine exposure status on the basis of their experiences of weight loss, hunger and cold during the 1944–1945 winter. There was some indirect evidence that the individual famine scores were accurate: the proportion of severely famine-exposed women increased with the degree of residential urbanisation during the famine, reflecting differences in severity of the famine between rural and urban areas (Burger *et al*, 1948). Since famine scores were based on recall, misclassification is a concern. However, we believe that this misclassification is unlikely to be related to the hormone concentrations under investigation, and, if anything, would result in an underestimation of the observed effects.

Our findings may be explained by long-lasting effects on the hypothalamo–pituitary–gonadal axis, although adrenal involvement cannot be ruled out. Contrary to our hypothesis, the direction of the effects would imply an increased breast cancer risk in nulliparous, famine-exposed women.
